# A Qualitative Exploration of the Role of Culturally Relevant Social Prescribing in Supporting Pakistani Carers Living in the UK

**DOI:** 10.1111/hex.70099

**Published:** 2024-11-10

**Authors:** Sarah McMullen, Shoba Poduval, Megan Armstrong, Nathan Davies, Chris Dayson, Kalpa Kharicha, Pushpa Nair, Manoj Mistry, Madiha Sajid, Kate Walters, Abi Woodward

**Affiliations:** ^1^ Research Department of Primary Care and Population Health University College London London UK; ^2^ Wolfson Institute of Population Health Queen Mary University London London UK; ^3^ Advanced Wellbeing Research Centre Sheffield Hallam University Sheffield UK; ^4^ NIHR Health & Social Care Workforce Research Unit, Kings College London London UK; ^5^ Patient and Public Involvement Partner

**Keywords:** culturally relevant, family carer, health and well‐being, health inequalities, Pakistani, social prescribing

## Abstract

**Background:**

Approximately 5.7 million people in the UK are providing informal care. Carers across all ethnic groups can experience negative impacts on their physical and mental health but some minority ethnic groups face greater challenges. Higher levels of social isolation exist among Pakistani carers compared to White British carers, yet the needs of Pakistani carers and how well support services meet these needs is less well understood. Social prescribing can help people get more control over their health care in a nonmedical way. South Asian and other ethnically diverse populations are under‐served in social prescribing and there is little evidence available on why this is the case.

**Aim:**

To explore the potential role of culturally relevant and adapted social prescribing in assisting Pakistani carers and identify the cultural and religious influences and barriers on carer health behaviours.

**Methods:**

Semi‐structured one‐to‐one interviews with Pakistani family carers (*n* = 27) and social prescribing stakeholders (*n* = 10) living in London and Sheffield, UK. Participants were recruited through voluntary and community sector organisations (VCSOs), social media, religious organisations, and word of mouth. Interview data was analysed in NVivo using reflexive thematic analysis methods.

**Findings:**

Two themes were developed; (1) Individual and community level influences: Navigating and accessing carer support within Pakistani communities, including carer identity and cultural barriers to accessing support provision, and cultural adaptation to facilitate support for Pakistani carer health and well‐being, (2) societal and structural level challenges: Accessing and providing social prescribing for Pakistani carers, including funding challenges for the provision of culturally relevant carer support, integration of primary care and social prescribing, and enhancing cultural awareness and competence in social prescribing for Pakistani carers.

**Conclusions:**

There are complexities surrounding carer identity, family dynamics, stigma, and a lack of knowledge of social prescribing within Pakistani communities. There is a need for more culturally competent support, culturally relevant education, awareness‐raising, and collaboration among primary care and VCSO's to better support Pakistani carers through social prescribing, which acknowledges and addresses the complexities.

**Patient and Public Contribution:**

The study included a patient advisory group comprised of two South Asian family carers who contributed towards all stages of the study. They provided feedback on study documents (topic guides and recruitment materials) and recruitment strategy, supported with translation of study documents and interpretation of the interview data, and helped with facilitation of our dissemination activities.

## 1 Introduction

Approximately 5.7 million people in the UK provide informal care; representing around 9% of the population [1, 2]. Informal care is unpaid care provided by a family member or friend to someone who cannot manage without their support due to physical/mental illness or disability [3]. Caregiving can negatively impact carers' physical and mental health across all ethnic groups, but it is important to understand the wider determinants of health that create greater health disparities in minority ethnic groups [4, 5].

In England and Wales, nearly 3% of the carer population is Pakistani [6]. Within Pakistani Muslim culture, caring for an elderly relative, which often falls upon women, connects with the ideal of showing respect for one's elders which is attached to the idea of family hierarchy, reputation and honour [7]. Many people from a Pakistani background are said to be in ‘deep poverty’ which occurs among people with less than 40% of median income after housing costs [8], having a compounding effect on health inequalities [7]. Higher levels of social isolation exists among Pakistani carers compared to White British carers, yet they are less likely to engage with mainstream support services including health and social care [9, 10]. A reliance on informal support within Pakistani communities due to cultural/religious norms reinforces familial traditions surrounding caregiving [11, 12]. Increased isolation and lower engagement with services may be worsened because of poor English language skills, gaps in awareness and understanding of services, conflicts with religion and culture, and issues around the cost of travel to attend appointments [13].

Healthcare services need to be delivered with an understanding of the cultural and religious influences within Pakistani communities. Many minority ethnic carers have a limited understanding of available support services within the UK [14]. Social prescribing is an all‐age, whole population approach. It is a nonclinical, multi‐stage intervention where referrals that typically come from professionals working in primary care, offer access to support, usually provided through local voluntary and community sector organisations (VCSOs). The types of support available to informal carers may be generalised or aimed at carers [15]. Social prescribing initiatives exist in 17 countries, but inconsistent definitions have led to a lack of robust evidence. Social prescribing recently has been defined as connecting individuals with community‐based nonclinical support to enhance health, well‐being, and community connections, and it is hoped to assist practitioners and policymakers internationally in fostering common understanding of the concept [16].

Within the UK, social prescribing is offered through the National Health Service (NHS). Models vary due to commissioning, funding, referral pathways, providers, and the VCSO infrastructure [17]. Social prescribing can offer economic benefits to primary care by reducing health and social care use [18]. Evidence from the National Academy for Social Prescribing (NASP) indicates South Asian and ethnically diverse populations are under‐served in social prescribing, highlighting that we need to understand why this is the case [19]. NASP suggest that to support social prescribing for people from ethnically diverse groups, communications/awareness raising, understanding cultural expectations, building trust, inclusivity, and outreach are of upmost importance. The role of social prescribing in supporting Pakistani communities is therefore under‐explored. A robust evidence base that includes multiple perspectives from individual, community and societal standpoints is crucial to implement changes and identify challenges relating to carer well‐being early on. This is especially important among a group that face cultural barriers to accessing services as well as being reportedly less trusting of healthcare professionals. Barriers include a lack of understanding of Asian culture, predominantly White providers, issues with interpreters, and fear of gossip [20].

This paper aims to explore the potential role of culturally relevant and adapted social prescribing in assisting Pakistani carers and identify the cultural and religious influences and barriers on carer health behaviours.

## 2 Methods

### 2.1 Design

This was a qualitative study using semi‐structured interviews. Taking a semi‐structured approach allowed for flexible and open‐ended questions that were non‐leading to build rapport with interviewees and minimise power imbalance between parties [21]. The study uses an experiential approach to qualitative research, focusing on understanding phenomena through individuals’ experiences within their social and cultural contexts [22].

### 2.2 Study Population and Setting

To make best use of existing community networks that the research team has with organisations supporting Pakistani communities, the setting for the study was London and Sheffield, England, with a focus on residential areas with high Pakistani populations. Participants eligible for the study were Pakistani family carers and social prescribing stakeholders (see Table 1).

**Table 1:** Participant inclusion criteria.

Inclusion criteria for carers	Inclusion criteria for stakeholders
Family carers aged 18+ years. Identified as being from the Pakistani ethnic group. Had current or recent experience (within the last 12 months) of being a main/primary carer for a family member. English and Urdu speakers	General Practitioners (GPs) and primary care healthcare professionals (e.g., occupational therapists/nurses). Social prescribing link workers. Representatives from voluntary and community sector organisations who provide social prescribing services.

**Exclusion criteria for carers**	**Exclusion criteria for stakeholders**
Carers under the age of 18 years. Those who lacked mental capacity to consent to the study.	Stakeholders with no experience of making or receiving referrals for social prescribing programmes among minority ethnic groups.

Interviews were conducted according to participants' preference (e.g., face‐to‐face or remotely via Zoom/MS Teams/telephone). S.M. is bilingual, speaking both English and Urdu. Carers were given the option to do the interview in either language.

### 2.3 Sampling and Recruitment

To achieve diversity in carer characteristics we purposively sampled for English/non‐English speaking carers, a mix of male and female carers, and those who had current and previous caring experience. Purposive sampling was supplemented with snowballing techniques [23]. Recruitment posters in English and Urdu assisted with identifying participants in London and Sheffield only. As many carers do not identify with the term ‘carer’, we used the wording ‘Do you care for a family member who cannot manage without your help’ in the poster. Translation of documents were supported by a Public Advisory Group (PAG). The study team approached over 50 organisations/groups (both new and existing contacts) to assist with recruitment and promoted the study through libraries, carers' centres, VSCOs/faith/cultural groups and social media (e.g., Twitter and WhatsApp)/newsletters. Stakeholders were recruited through VCSOs, professional bodies and social media (e.g., Twitter and LinkedIn). Tables 2 and 3 detail participant characteristics.

**Table 2:** Carer characteristics.

Category	Number of participants (%)
Recruitment location	
Greater London	17 (63%)
Sheffield	10 (37%)
Gender	
Male	8 (30%)
Female	19 (70%)
Average age (range)	44 years (18–63)
Interview language	
English	20 (74%)
Urdu	7 (26%)
Interview method*	
Video conferencing (Zoom/MS Teams)	21 (78%)
Telephone	5 (18%)
Face to face (participants own home)	1 (4%)
*Length up to 90 min (mean time 66 min)	
Employment status	
Employed part‐time	7 (26%)
Employed full‐time	3 (11%)
Unemployed (including looking for work)	17 (63%)
Average household size (range)	4 (2‐10)
Relationship with the person/s being cared for (includes those caring for more than one person):	
Parents and in‐laws	17 (55%)
Spouse	5 (16%)
Children (including adult children)	7 (23%)
Sibling	1 (3%)
Extended Family	1 (3%)
Reported health condition/s of the person being cared for (includes those with multiple ‐long‐term conditions):	
Cardiovascular disease	12 (23%)
Immobility	8 (15%)
Arthritis	7 (13%)
Dementia	6 (11%)
Cancer	6 (11%)
Learning disability and/or difficulty	5 (9%)
Mental health conditions	5 (9%)
ADHD and/or Autism	4 (8%)
Hours per week caring	
<35 h	2 (7%)
>35 h	25 (93%)
Receiving carer's allowance (non‐means tested benefit)	
Yes	11 (41%)
No	16 (59%)

**Table 3:** Stakeholder characteristics.

Category	Number of participants (%)
Stakeholder group	
Social prescribing link worker (SPLW)	5 (50%)
Healthcare professional (General Practitioner)	2 (20%)
Healthcare professional (Occupational therapist)	1 (10%)
Voluntary/community sector providers of social prescribing	2 (20%)
Recruitment locations	
Greater London	6 (60%)
Sheffield	4 (40%)
Gender	
Male	4 (40%)
Female	6 (60%)
Interview method	
Video conferencing (Zoom/MS Teams)	10 (100%)
Ethnicity (as self‐defined by stakeholder)	
Pakistani/British Pakistani	4 (40%)
White British	3 (30%)
South Asian	1 (10%)
Yemeni	1(10%)
European	1 (10%)
Experience of referring *carers* or receiving referrals for *carers* for social prescribing programmes (*n* = 9)	
SPLW	4 (44%)
Voluntary/community sector providers of social prescribing	2 (22%)
Healthcare professional	3 (33%)
Experience of working to support people from a *Pakistani background* (past or current) (*n* = 8)	
SPLW	3 (38%)
Voluntary community sector rep	2 (25%)
Healthcare professionals	3 (38%)

### 2.4 Data Collection

Between March and July 2023, SM conducted interviews with Pakistani carers (*n* = 27) and stakeholders (*n* = 10). Participants provided written/verbal (audio recorded) informed consent. Separate interview topic guides for carers (e.g., covering carer role/identity, health and well‐being, access to current support, additional support needs), and stakeholders (including a shortened version for healthcare professionals (HCPs) to accommodate time constraints), were collaboratively developed with the research team and PAG. Participants were provided with a participant information sheet and offered the chance to ask questions. Carers received a £50 voucher for participation, as outlined in study documents. The amount was recommended by the PAG. For Urdu‐speaking participants study documents were orally translated into Urdu by S.M. and M.S. All interviews were audio recorded. Each participant completed a short demographic form before the interview.

### 2.5 Data Management and Analysis

Audio recordings were transcribed verbatim, checked for accuracy, and anonymised. Urdu recordings were translated and transcribed by S.M. Transcripts were imported into NVivo and reflexive thematic analysis (RTA) was used [22]. In RTA, themes are actively constructed by the researcher, shaped by the data and the researcher's interpretive framework, including their assumptions. Reflexivity is crucial for effective thematic analysis, involving reflection on the researcher(s)' assumptions and biases. The researchers in this study reflected on their positionality, power and beliefs about caregiving and Pakistani culture to ensure the analysis was shaped by participants' lived experiences, not their own assumptions. Involvement of a PAG assisted further with the validity of data, that is, whether interview transcripts are deemed an accurate representation (see example of PAG perspectives on analysis in Supporting Information: Appendix 1). Initial coding was created by S.M., themes were developed in consultation with A.W. and PAG and reviewed by the team. S.M. is an early career researcher from a Pakistani background. A.W. is an experienced qualitative researcher and led the project. The multidisciplinary research team comprised of experts within primary care, psychology, sociology, epidemiology and health inequalities. Involvement of a PAG and multidisciplinary team allowed for the positionality of the researcher (i.e., their own power, privileges and biases) to be acknowledged and challenged during the development of themes and analysis. The analysis draws upon a socio‐ecological model to present participant experiences and allows for an in‐depth exploration of the interplay between individual, community, and societal factors [24]. Taking a social ecology approach allows for a better understanding of the different levels of influence on health behaviours/outcomes and trust, and to better understand how structural challenges/barriers interact with individual factors [25]. By considering the multiple levels of influence, a socio‐ecology model promotes a holistic approach to addressing complex issues, leading to more effective and sustainable solutions.

## 3 Findings

The findings highlight nuanced examples of how culture, religion and gender‐norms intersect to influence carer roles, identities, and health behaviour and outcomes. The concept of social prescribing as an existing community‐based intervention is explored as a potential way to assist Pakistani carers. Table 4 illustrates the themes that were developed from the data.

**Table 4:** Themes.

Themes	Sub‐themes	Description
Individual and community level influences: Navigating and accessing carer support within Pakistani communities	Carer identity and cultural barriers to accessing support provision Cultural adaptation to facilitate support for Pakistani carer health and well‐being	Explores cultural and gender norms around caregiving that hinder access to support, including reluctance to seek help. Highlights the benefits of culturally adapted and gender‐sensitive services.
Societal and structural level challenges: Accessing and providing social prescribing for Pakistani carers	Funding challenges for the provision of culturally relevant carer support Integration of primary care and social prescribing Enhancing cultural awareness and competence in social prescribing for Pakistani carers	Explores the structural challenges to the provision of culturally tailored carer support/social prescribing, and the role of cultural competence at the societal level. Key challenges focus on funding constraints across the voluntary and community sector and integration of primary care and social prescribing services.

The data extracts below include insights from male and female carers (e.g., C01) and social prescribing stakeholders (e.g., S01). As detailed in Table 2, more than half of carer participants were looking after a parent/in‐law and nearly all were caring for a family member for more than 35 h a week. It was most common for carers not to be in paid employment alongside their caring role. All carers were under the age of 65, with the average age being 44 years.

### 3.1 Individual and Community Level Influences: Navigating and Accessing Carer Support Within Pakistani Communities

#### 3.1.1 Carer Identity and Cultural Barriers to Accessing Support Provision

Most carers saw caregiving as their ‘duty’ and taking care of their loved one was often not a choice but a responsibility they were born into. As such, carers highlighted variances around self‐identifying as caregivers, with a small number avoiding the label altogether:

“I think what needs to be recognised is…there's a lot of people that don't refer to themselves as carers and I think there's a lot of stigma attached…” *(C11_43yrs_female)*

Like other carer groups, Pakistani carers consistently prioritised others' needs over their own, leading to sacrifices in daily life. Looking after family members who are in need, sick or elderly was often a cultural expectation, particularly for Pakistani women. Many female carers could therefore find it difficult to navigate support to benefit their own health and well‐being needs:

“Depending on my good days and bad days, I am more, “Oh my God,” you know, I resent it…I also resent myself for feeling like that…I was brought into this world because…the daughter's role was to watch, to care for my mother when she gets old…some days I feel like I am fulfilling the reason that I was brought into this world. Other days, it's like I want my own life… And the reason why I…have done so much myself rather than getting it outsourced, that is my own, how would I sleep at night?” *(C06_41yrs_ female)*

Duty of care was culturally/religiously influenced within Pakistani families, with caring responsibilities being predominantly a female role:

“I think a lot of it is male dominance…it's a woman's role to still take care of people. I'm angry about that…and the male members of the family not taking responsibility.” *(C07_63yrs_female)*

Challenges around family conflict and caregiving were commonplace since getting access to support from outside the home was typically a decision made by the family unit. External support was minimal due to stigma and misconceptions around entitlement or types of support:

“I don't think the caring externally from organisations…or Council…[is] going to work because there's too much stigma attached to it…I just don't think culturally that would be allowed…I know what we're entitled…but it's not something we would do, the family is too proud.” *(C11_41yrs_female)*

Carers who experienced internal feelings of stigma or being judged from family for needing additional help with caregiving were less likely to seek support:

“…I cannot share anything with my parents [about my caring role], they will feel like I have failed as a mother…I don't even meet people because people judge me, so no one comes to our house to visit…I don't meet anyone because of my children. Our Pakistanis do not know about Autism and ADHD, they are too far behind. To relax I speak to my sister…every day. She lives in Pakistan, but I haven't even told her about my children.” *(C04_43yrs_ female_Urdu)*

Carers also revealed that the person registered with a General Practitioner (GP) as the caregiver, may not always be the primary caregiver. Participants preferred to avoid family conflict over informing a GP of their role; potentially limiting HCPs' ability to offer support such as through a social prescribing referral. Carers seldom visited a GP for themselves and typically associated seeing a GP with their physical health and being ‘sick’:

“I am not sick; I think I only go to the GP when I'm having symptoms, and I can't get drugs over the counter. So, I don't know how to benefit from the GP.” *(C01_23yrs_female)*

Among carers who did visit their GP, a small number faced challenges when their GP was from the same background as assumptions could be made about cultural lifestyle and reliance on familial networks:

“I feel they [South Asian doctors] don't take mental health seriously. As my GPs are all English, so when I mentioned it, she understood what I was going through…what kind of help I need. In terms of Asian doctors…you have to cope with it. Because they feel Asian families like tight knit…so they are support for each other.” *(C02_52yrs_female)*

Both stakeholders and carers highlighted the importance of addressing language barriers within support systems such as social prescribing and having a good grasp of culture, as explained by a social prescribing link worker (SPLW) below:

“I think there's barriers to communication …if someone's not speaking the language or understanding the demographics…it's already going to stop that person from saying what they need or want…If you put everybody in one room and you've got Pakistani carers and…you've got other carers…they might be too embarrassed to share…They won't relate, and they won't feel like it's for them, so they won't engage.” *(S03_British_Pakistani_SPLW)*

Support services were considered too generalised without taking cultural and religious values into account:

“…a lot of the stuff out there, it's not relevant to something that I can relate to, or my family can relate to. I know a lot of friends and family are in the same boat…. it's just like give us something that would cater to our needs in terms of culturally or religiously” *(C12_36yrs_male)*

In summary, cultural barriers at the individual and community level included carer identity and duty of care, terminology/language, stigma around seeking additional support. These could lead to experiences of exclusion and people feeling judged.

#### 3.1.2 Cultural Adaptation to Facilitate Support for Pakistani Carer Health and Well‐Being

Few carers accessed tailored carer support, with female carers emphasising the importance of women‐only activities, like swimming, and gender‐specific emotional support due to cultural and religious norms. Certain services or activities were deemed culturally appropriate for women‐only sessions, while other activities could be mixed:

“It's OK for men and women to be together…but it also depends on what we're talking about and activities. Some things might be inappropriate to speak of in front of women and they also might not want to share any personal information with [men].” *(C27_50yrs_male_Urdu)*

One SPLW who worked within Pakistani communities, explained it is possible to develop culturally competent support and adapt existing services, but more tailoring of this kind is needed more broadly:

“…I've helped develop…a South Asian seniors' group for men, separate. A South Asian group for women, separate…Then there's a women's group for all ages 16 and above…Then there's Islamic counselling…a bit of fitness…It's about well‐being. [The organisation] kind of adapted and we feed into that when we speak to them” *(S03_British_Pakistani_SPLW)*

Sessions run through Mosques could help people connect with their religion and facilitate social interaction with others:

At least three times a week we'd be going from one Mosque for another programme of some sort to get [mum] out and about… We lost all of that in COVID and it's never come back… Because that was my mum's social interaction and that's where I took my mum, that was my social interaction as well… we have become isolated from the community… *(C06_41yrs_ female)*

Having social prescribing services that were provided in culturally or religiously appropriate settings such as Mosques was therefore paramount due the significant role that religion plays in connecting people:

“Yes, massively religion plays a massive, massive role…When you're thinking about social prescribing and reconnecting people to community one of the biggest wins you can get is having someone reconnect with their religion because that's such a great way to find a community with something in common, something that you understand, and it has the values…” *(S05_White_British_SPLW)*

Improved communication, messaging and awareness raising of the support available to Pakistani carers were highlighted by carers and stakeholders. A small number of carers had heard about or understood the concept of social prescribing due to links with their employment but none of the carers had received a social prescribing referral. The majority of carers did not understand what the term ‘social prescribing’ meant, and many had not heard of the term before. However, upon explaining what social prescribing is during the interviews, the response among carers regarding the potential benefits was extremely positive:

“No, I haven't heard of that, but it sounds good. It would be nice to have some support like that from someone.” *(C04_43yrs_ female_Urdu)*

While very few participants were accessing carer‐specific support, those who were, saw the benefits such as the participant below who used money accessed through a carers' grant to take time out and focus on their own well‐being:

“…they have…[a carers fund] where you can apply for…then use that money and go out as a family…I've been using that money for my health reasons. My own health [is] deteriorating so I've been using that for massage…” *(C02_52yrs_female)*

The few carers receiving support, accessed it primarily through VCSOs, including South Asian/religious groups offering community activities, exercise, and advice. These groups were popular due to shared backgrounds among attendees and culturally aligned activities.

Groups such as the South Asian women's group accessed by one participant were not specifically set up for carers but inadvertently provided much needed peer support as they were often frequented by other female carers:

“I don't do anything to take care of myself and my health or well‐being…[Cultural Organisation] used to ask me to attend her [South Asian women's group] because they are near our house, so at least if go there I get some relief [from caring role]” *(C04_43yrs_ female_Urdu)*

For a small number of carers, seeing a GP from the same background who may also speak Urdu was a facilitator of support. Having a good relationship with a GP could be reassuring due to shared cultural understanding which fostered a sense of comfort, allowing them to be more open to accessing support within their caregiving role:

“My GP is Pakistani, and they understand what I'm going through, what's our culture, our religion, what our family values are like so that's really helpful. We used to go to a white GP before and it was very difficult, the language barrier was a big problem and most of the time there was no interpreter…it's still a big communication issue.” *(C08_41yrs_male_Urdu)*

While the above quote presents a contrasting view from a previous point surrounding stigma and mental health, these excerpts serve to highlight the importance of trust in building effective relationships, both with healthcare systems and communities.

There is shown to be a lack of culturally relevant provision that focus on the needs of Pakistani carers. Existing cultural provision could be adapted for carers and similarly, there are opportunities for existing carer services to be culturally adapted. These would need to consider gender sensitivities, connections to faith and culture, and aimed at tackling key issues facing Pakistani carers such as experiences of isolation.

### 3.2 Societal and Structural Level Challenges: Accessing and Providing Social Prescribing for Pakistani Carers

#### 3.2.1 Funding Challenges for the Provision of Culturally Relevant Carer Support

Social prescribing programmes rely heavily upon VCSOs as service providers of support, yet stakeholders emphasised the challenges they face in the current economic climate:

“The voluntary sector always need help in funding. That way they can deliver more, employ more staff, develop more in the organisation to support the community that they need to support.” *(S06_South_Asian_SPLW)*

Funding constraints hindered the optimal level of tailored support that VCSOs could provide to Pakistani carers through social prescribing pathways. Disparities across support could be very localised and VCSOs serving communities within areas of high‐deprivation struggled with funding that was short term, had run out, or not available for specific groups:

“…most [Pakistani women]…are looking for swimming sessions, we can't afford it as [a VCSO]. We have no fund for it…before in 2014, yes, there is a bus which takes the ladies to the swimming pool but there is no fund, we can't carry on with these.” *(S01_Yemeni_SPLW)*

Carers also highlighted that many groups, including a former carers group run by a South Asian VCSO, had ceased due to funding ending. They too, highlighted that localised funding constraints restricted the availability of culturally relevant services:

“The local Pakistani ladies have tried to set up a group in [area], but we don't [have] any funding…We spoke about this to the local councillor, but she said…that for funding we will need to set it up as a charity…We are Pakistani, we can't speak or read English very well. How will we do this?” *(C04_43yrs_female_Urdu)*

Funding constraints significantly limited the ability of VCSOs to provide tailored support for Pakistani carers through social prescribing. It could be frustrating for VCSOs and link workers who wanted to make a difference by going beyond the existing provision for carers:

“When is it going to change? When are you going to say, ‘Here you go, we're going to hand this to you and this is what you want. We want you to replicate [registered carers charity], but we want you to do it for the Pakistani community…unity.’ I've sat on panels, I've sat on boards, inequality boards. I've done it all, and really even me – and I've got a loudmouth – I can't even make an impact and a difference.” *(S03_British_Pakistani_SPLW)*

Financial challenges hindered the development of culturally relevant services, exacerbating the difficulties faced by Pakistani communities who predominantly live in high‐deprivation areas.

#### 3.2.2 Integration of Primary Care and Social Prescribing

As identified previously, carers accessing primary care faced challenges during health consultations which could impact their access to crucial support, such as through social prescribing which relies significantly on GP referrals. While it was less typical for carers to see a GP about their own health and well‐being needs due to prioritising the person they care for, those who did seek help were not always offered support, as one carer shared:

“There were no like putting me in touch with a social prescriber or anything like that…since I've learnt about a social prescriber, I'm very shocked that I wasn't…I think at the lowest point that I was, for the doctor to do anything about it, I was quite shocked about that.” *(C11_41yrs_female)*

This illustrates the perceived lack of appropriate support after raising concerns with the GP. Despite the growing recognition of social prescribing as a tool for addressing non‐clinical needs, none of the carers in this study reported receiving a referral from their GP for such services.

Barriers to accessing support included both low help‐seeking behaviour among carers and obstacles within the social prescribing referral pathways. As one GP noted, a key challenge was around feeling confident that social prescribing would result in appropriate support:

“So, often we went to social prescribing…because we feel so impotent… But what is missing there? Is it cultural or is it that they're not culturally specific enough? I think it's the second, in my opinion.” *(S09_British_Pakistani_GP)*

Some HCPs expressed a desire to use social prescribing more effectively but had experienced frustration of the tendency to treat social prescribing as a transactional process as opposed to a more patient‐centred approach:

“[Link workers] just action referrals and signpost… I think that's a big failing of the social prescribing thing. We don't work like that in primary care. We follow through… I barely know what my social prescriber looks like…She's a name on a clinic list…” *(S08_Pakistani_GP)*

The data presented highlights a lack of GP referrals when carers reach out for support which may be due to lack of integration, pointing towards a need for better integration between GPs and social prescribing services in general.

#### 3.2.3 Enhancing Cultural Awareness and Competence in Social Prescribing for Pakistani Carers

Nearly all carers said they would go to their GP and ask about social prescribing following the interview, suggesting that more engagement/outreach with carers is needed. To enhance awareness of the benefits that social prescribing can have for carers from diverse backgrounds, it was recommended that changing some terminology used to describe services would be helpful. The meaning behind the term ‘social prescribing’ was unclear for many carers and stakeholders suggested that terms like 'respite' may cause confusion or be a barrier for carers seeking support:

“There's a negative connotation to [respite]…for the Pakistani community if somebody doesn't even know what respite means, and their daughter explains what respite means…they'll be like, well…sounds a bit like weird…I would use a positive thing like self‐care break or something in Urdu…that's universal, like ‘Khushi [happiness]’ it's Punjabi…its Urdu as well.” *(S03_British_Pakistani_SPLW)*

Stakeholders suggested that more information in written and verbal formats to align with different preferences for learning about available support would be beneficial:

“I think [carers] need more information…I think discussions are probably better than literature…I don't know if they'll read it, or they'll get the time. Talking to these people about it and letting them know is probably a key.” *(S06_South_Asian_SPLW)*

Carers suggested various advertising methods that they thought would enhance awareness of social prescribing opportunities beyond healthcare facilities such as using social media (e.g., WhatsApp and Facebook), having materials in Urdu, and collaborating with cultural/religious organisations or carers charities to share information:

“WhatsApp groups, they have events on there…secondly…Facebook, and emails…personally I don't really go to the GP that much so if somebody is going regularly to the GP then they'll be able to access it but other than that…I think what's good in that sense is the carers newsletters.” *(C02_52yrs_female)*

Social prescribers are required to take a proactive approach to come up with tailored plans that address individual needs but as highlighted earlier, identifying carers can be challenging. As such, any mechanism that encourages the identification of Pakistani carers would likely lead to more effective and proactive social prescribing:

“…if people were identified as carers, we could run a list and target…do proactive social prescribing with them…But…if they're not identified as carers that's the first step we need to take.” *(S03_British_Pakistani_SPLW)*

To support with proactive social prescribing within Pakistani communities, several stakeholders highlighted the importance of specific training, emphasising that cultural awareness does not solely depend on belonging to the same culture or ethnic group.

“It doesn't have to be someone from the same culture…just…someone who totally understands where they're coming from and understands their hesitance, their resistance, their cultural values and works with those.” *(S09_British_Pakistani_GP)*

The above point was reiterated by a White British HCP who explained how they tried to educate themselves in taking a culturally sensitive approach to supporting patients within the social prescribing pathway:

“…I've made a conscious effort to network shamelessly…also, I think knowing‐learning about the patient group here. I don't come from a Pakistani background… in terms of being like culturally sensitive…I wanted that information…[how] I can best support my patients in terms of the [social prescribing] pathways…” *(S07_White_British_Occupational_Therapist)*

To summarise, improving cultural competence and targeted outreach could assist in enhancing the accessibility and effectiveness of social prescribing for Pakistani carers. This may be achieved by addressing language barriers, refining terminology, and increasing proactive identification of carers and support needs within Pakistani communities.

## 4 Discussion

The themes and sub‐themes presented support the level‐specific approach (individual, community, society) of the socio‐ecological model used and were developed from the interview data. The findings provide novel contributions to evidence about the accessibility of social prescribing for people from minority ethnic backgrounds to create a bigger picture of the awareness and use of social prescribing among Asian population groups [19]. The findings highlight how community and societal level challenges interact with individual‐level factors/influences relating to Pakistani carers' identity, wider family structures and gendered norms. Reports from carers and stakeholders provide evidence on the need for non‐clinical support that is culturally relevant and gender specific, which could be addressed through social prescribing. The preference within Pakistani communities for seeking informal support and the continuation of gendered roles within families [12] is compounded by the stigma and family conflict associated with seeking mental health support from external sources. These are complex areas that must be considered to better understand health behaviours and outcomes among Pakistani carers [26].

An overall lack of awareness of social prescribing among Pakistani carers, coupled with cultural challenges associated with the term ‘carer’, impacts on carer self‐identification, social prescribing referrals, effective identification of carers by HCPs, and accuracy of registered carer data among patients, which may not be unique to Pakistani communities. It is therefore important to acknowledge that while challenges associated with the self‐ and social identification of carers exist more broadly across ethnic groups and may be cross‐cultural, the nuances surrounding cultural norms, religion, gender and family dynamics need to be considered within the context of individual communities. Seeking help for caregiving is complex due to its often‐hidden nature and the influence of broader social and economic structures on individuals' health, combined with personal social and cultural factors [25].

Social prescribing is a key component of Universal Personalised Care in UK policy, aimed at addressing the shortcomings of ‘one size fits all’ services that fail to engage those most in need [27]. Personalised care enables patient autonomy over care planning, keeping in mind what matters to them the most [28] such as cultural aspects that may not have been addressed previously [29]. The Pakistani carers included in this study rarely visited their GP for their own needs, partly due to mistrust and the stigma around mental health in South Asian communities and families [20]. To encourage greater individual autonomy over carer health and well‐being within Pakistani communities, and more effective carer support at the societal level, it would be beneficial for HCPs to have improved understanding of cultural dynamics, like joint decision‐making in Pakistani families and South Asian family structures/practices [7].

Evidence shows that targeted social prescribing support tailored to specific individual needs (e.g., older people, those with long‐term conditions and their carers) [30], is beneficial, especially for individuals experiencing social isolation and mental health problems [18]. Where possible, carers in this study sought support in‐line with their cultural/religious requirements. However, the support available through cultural organisations was typically generalised rather than carer‐specific and similarly, the carer support on offer was not culturally relevant. The findings show that despite challenges around identification of carers (self and social), social prescribing programmes that are both tailored around carers and culturally relevant would be beneficial.

The findings from both carers and stakeholders indicate a need to reassess social prescribing pathways beyond primary care routes to address the under‐representation of Pakistani carers in referrals. In doing so, the lack of integration between primary care and social prescribing services within communities must be acknowledged to support more effective use of social prescribing among underserved populations. The lack of integration, combined with a shortage of culturally relevant support therefore continues to pose challenges for carers seeking help as well as those within primary care who are in a position to facilitate support.

The carers interviewed, often saw little benefit in disclosing their caring role with a GP but were positive about the future potential of social prescribing to support their health and well‐being. As discussed in an earlier section, involvement in this research led participants to organically state that they would engage more proactively with social prescribing, which in turn, would challenge pre‐existing mental health stigma within Pakistani communities. This highlights the necessity for targeted promotion and culturally relevant messaging within Pakistani communities about available support and a need for clearer terminology in relation to ‘social prescribing’. Effective communication strategies should extend beyond primary care, leveraging community channels to raise awareness and engagement with social prescribing services. Training for HCPs and social prescribers around cultural awareness/competence and service‐led co‐design of culturally tailored support programmes is recommended [31, 32].

Guidance on the delivery of proactive social prescribing which is advocated by NHS England and introduced as an additional requirement for primary care networks [33], would be beneficial. The provision of proactive personalised care and prevention is also of international importance within primary care, as outlined by the World Health Organisation [34]. It supports a targeted strategy to address disparities, improve health equity, and ensures that diverse populations, such as Pakistani carers, receive appropriate support and resources for their unmet needs.

**Figure 1:**
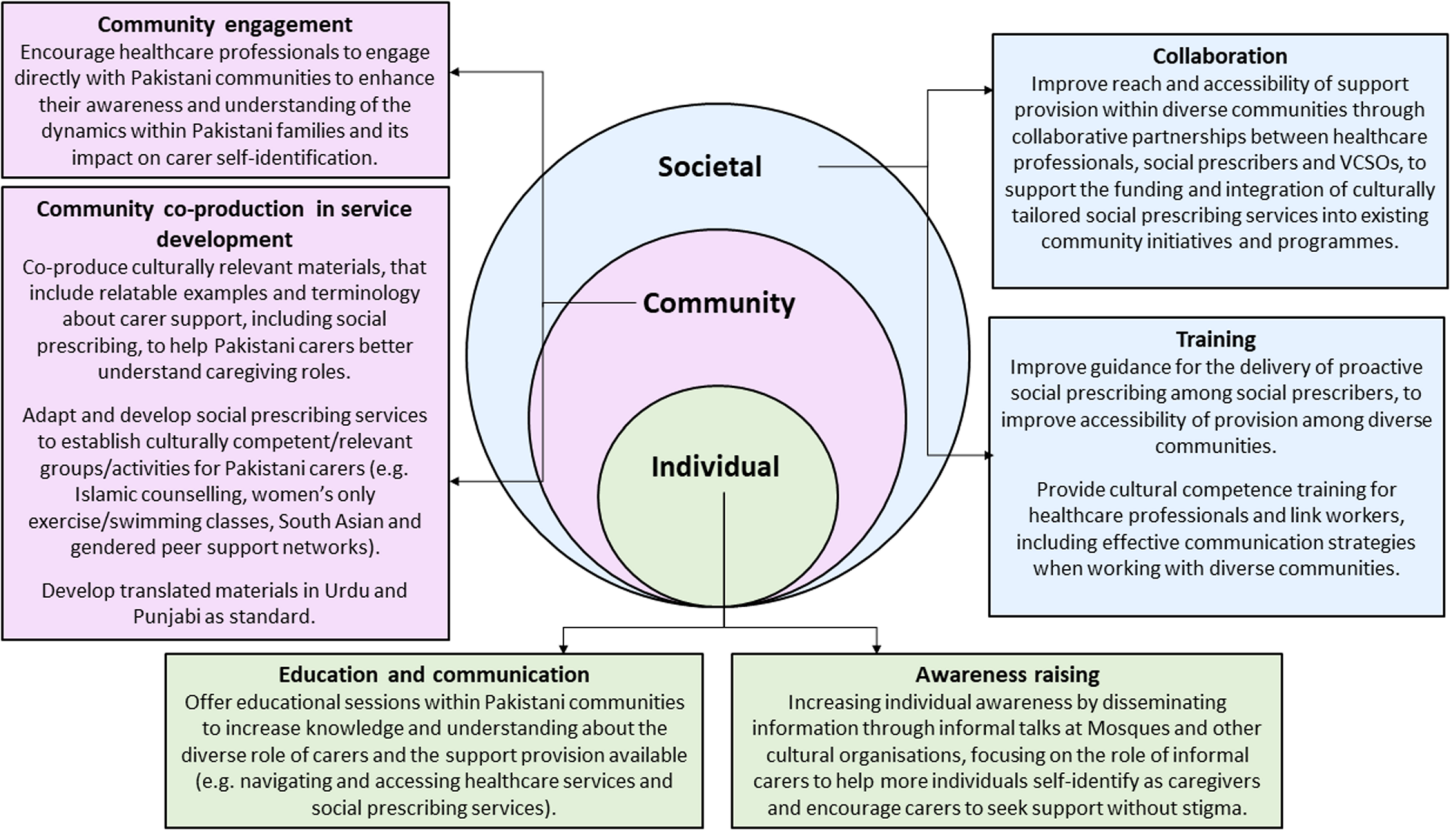
Recommendations to adapt, refine and improve social prescribing services for Pakistani carers.

Inclusive, ethnically diverse participation in proactive social prescribing development will promote holistic, individualised care that addresses the social determinants of health [34]. The findings provide a significant contribution to evidence on the importance of tailored and culturally relevant approaches to social prescribing. The data challenges critics who argue that healthcare services, including social prescribing, should be universally designed to avoid fragmentation and that a cultural focus could complicate implementation and effectiveness [35]. To achieve a model of social prescribing that is beneficial to Pakistani and other minority ethnic carers, these structural and societal challenges need to be addressed, along with the barriers to investment in community‐based services and funding limitations faced by VCSOs (see recommendations in Figure 1) [36].

Alongside recommending culturally relevant social prescribing programmes, it is important to acknowledge that social prescribing is just one way that Pakistani carers can potentially be assisted as it is an intervention that already exists and can be adapted/tailored with communities. Pakistani and South Asian communities are not homogenous and social prescribing may not be right for everyone. Other options for supporting the health and well‐being of Pakistani carers could include provision directly offered by faith/community‐based organisations. Nevertheless, addressing the inequalities highlighted, requires co‐production; 'an approach to working together in equal partnership and for equal benefit' [37]. Successful social prescribing programmes and other community‐based interventions are those that are designed together with HCPs, link workers, VCSOs and individuals/service users (see system‐level recommendation in Figure 1). Fostering community ownership of support services is essential in strategic co‐production of culturally adapted and relevant services for diverse populations. Interventions that are not designed or tested within these populations may result in less effective support services, potentially widening the health inequalities gap [28].

## 5 Implications for Research, Policy and Practice

The findings and recommendations from this study will interest researchers, policymakers, and practitioners focused on health equity in Pakistani and South Asian communities. While the economic contribution of family carers to the NHS is recognised [38], they remain a hidden population. It's crucial for policy and practice to appreciate carers' dedication and promote workforce diversity to address health inequalities holistically.

Figure 1 presents our data‐driven recommendations based on the socio‐ecology approach, addressing individual, community, and societal influences on health behaviour. These recommendations highlight the need for more funding, increased partnership, and co‐production to support culturally relevant social prescribing for Pakistani carers, considering culture, religion, and gender norms [25].

Future research with South Asian communities can benefit from the inclusive approach taken in this study, particularly in engaging carers who do not self‐identify with the ‘carer’ label. Building on this work, future studies should explore the barriers and facilitators of social prescribing services for South Asian and other minority ethnic groups more broadly.

## 6 Strengths and Limitations

A main strength is the ethnicity of the chosen study population, which is underserved, as well as the inclusion of Urdu speakers and Pakistani male carers who are seldom heard. The study addressed a gap in evidence relating to the accessibility of social prescribing among South Asian groups, contributing to knowledge and understanding about why UK Pakistani communities are under‐represented in social prescribing. Internationally, the findings will be of interest to the International Social Prescribing Community of Practice [39], providing opportunities on a global scale to promote more culturally relevant and tailored programmes of support.

One potential limitation of the study is that data was collected from only two UK cities. Social prescribing services differ nationally and internationally meaning the geographical transferability of data needs to be acknowledged. The study inadvertently did not include carers who had accessed social prescribing. It also did not capture experiences of those from other South Asian groups. The perspectives of older generation carers (e.g., above 65), who may have different support needs/face additional challenges due to their declining health, were not represented.

## 7 Conclusion

This research revealed complexities surrounding carer identity, family dynamics, stigma, and a lack of awareness of social prescribing within Pakistani communities. Our findings emphasised the importance of raising awareness about social prescribing services and support for carers amongst Pakistani communities, emphasising that cultural competence needs to be at the top of the agenda for delivering support to Pakistani carers, and that greater collaborations are needed across community/religious organisations, health care, and the voluntary/community sector. This includes finding opportunities to culturally adapt existing carer‐specific social prescribing programmes as well as tailoring existing support offered by cultural organisations to directly address the needs of Pakistani carers. As such, health care, social prescribing and other support services for Pakistani carers need to be delivered with the complexities surrounding specific cultural and/or religious influences and the gendered roles attached to caring responsibilities in mind.

## Author Contributions

**Sarah McMullen:** formal analysis, writing–original draft, writing–review and editing, investigation, data curation. **Shoba Poduval:** writing–review and editing. **Megan Armstrong:** writing–review and editing. **Nathan Davies:** writing–review and editing. **Chris Dayson:** writing–review and editing. **Kalpa Kharicha:** writing–review and editing. **Pushpa Nair:** writing–review and editing. **Manoj Mistry:** writing–review and editing. **Madiha Sajid:** writing–review and editing. **Kate Walters:** writing–review and editing. **Abi Woodward:** conceptualisation, funding acquisition, writing–original draft, writing–review and editing, supervision, methodology.

## Ethics Statement

Ethical approval was granted by the University College London (UCL) Research Ethics Committee (22357/002).

## Conflicts of Interest

The authors declare no conflicts of interest.

## Supporting information

Supporting information.

## Data Availability

The data that supports the findings of this study are available in the supplementary material of this article.
