# Supplementary material for: A Qualitative Exploration of the Role of Culturally Relevant Social Prescribing in Supporting Pakistani Carers Living in the UK
Source: Health Expect. 2024 Nov 10;27(6):e70099. doi: 10.1111/hex.70099 (PMC11551476; doi:10.1111/hex.70099)
Supplement: Supplementary file 1 — Supporting information. [file HEX-27-e70099-s001.docx]

Interviewer: What do you think about your caring role and how was it decided that you would be the carer for your mum?

Respondent: It wasn’t really decided [**PAG comment: Lack of control over choices]**. It was just a case of our two sisters and I just started doing it. There was no conscious decision. It was just a case of I’ll just take over.  **[PAG comment: and unsaid assumptions]**

Interviewer: Do your sisters live with you or they’ve got – they –

Respondent: One of them does, the older one.

Interviewer: You said that this kind of just came upon you so do you think – do you see it as your duty to take care of your mum?

Respondent: Yes, most definitely. **[PAG comment:** **Strong sense of responsibility]**

Interviewer: Why is that and how does that make you feel?

Respondent: Why is an interesting question because it’s my responsibility to take care of my parents in their old age. I actually read a study about Chinese children taking care of their parents. There’s less mental anguish in having to do that because there’s a built-in assumption **[PAG comment: Yep!]** that we will look after our parents and grandparents in any case. Whereas in the West there’s more of a cognitive dissonance, so I don’t see it as any kind of issue **[PAG comment: Respondent displays a wider sense of differences between cultures]**

. I actually see it as a positive for society if you look after your parents. What was the second part of that question?

Interviewer: How does it make you feel? Just before you answer that, just unpick what you said. So, you mentioned about this Chinese study but do you think there’s aspects of being Pakistani or being Muslim that you also see as your duty?

Respondent: In both religion and tradition, both dictate that a person – a child should take care of his parents. To be fair with you, it’s a case of I’d like to take care of my parents because if I was in their – when I’ll be in that situation when we’re older and I need somebody to look after me, I’d rather my children as opposed to the state **[PAG comment: Does not want to be a burden on social services]**

looking after me. So, it’s both my duty and also a bit of selfishness as well in terms of setting that precedent.**[PAG comment: Role modelling for their own children]**

Interviewer: Why do you think having children look after you is better than having the state look after you?

Respondent: [Pause] I think it’s a fairly obvious answer, isn’t it? Would you rather a stranger who doesn’t really have any vested interest looking after you? Or would you want to be looked after somebody [sic] who you’ve spent your whole life nurturing and to a point where those roles are reversed? It’s an innate thing that you want to be looked after by somebody. You get married to somebody who you want to take care of and somebody who takes care of you. You want to have children to raise correctly and in your old age to help you. You want them to give you the honour of your old age.**[PAG comment: Does state not give that honour in the same way as children might do? This can be exactly opposite in many families where children have abandoned their parents, and State is looking after them.]**

Interviewer: How does it make you – the second part of my first question was –

Respondent: How does it make you feel?

Interviewer: Yes.

Respondent: I don’t actually have any real feelings about it. It’s a case of this is what I was always going to do. I don’t have any, “I wish I didn’t have to look after mum. I could just chuck her in a bin or an old people’s home and just go off to Majorca and do whatever I need to do.” I don’t have those wants or desires. It’s a case of it’s better for me. It feels correct. It feels right **[PAG comment: Strong sense of values]**. It feels comfortable for me to look after her. Don’t get it wrong, it is frustrating at – it does get frustrating **[PAG comment: Also acceptance that caring comes with its own challenges]**

when you have to repeat yourself over and over again on certain things which you think are quite simple. But you can’t expect somebody to do something that they can’t do. So, there are frustrations, of course, but no, it feels like it’s something I should be doing and something I want to do.
